# Detrimental Effect of Cannabidiol on the Early Onset of Diabetic Nephropathy in Male Mice

**DOI:** 10.3390/ph14090863

**Published:** 2021-08-28

**Authors:** Beatriz Carmona-Hidalgo, Adela García-Martín, Eduardo Muñoz, Isabel González-Mariscal

**Affiliations:** 1 Emerald Health Biotechnology, 14014 Córdoba, Spain; cahibeatriz@gmail.com (B.C.-H.); adelagarcia@emeraldpharma.life (A.G.-M.); 2 Instituto Maimónides de Investigación Biomédica de Córdoba, Departamento de Biología Celular, Fisiología e Inmunología, Universidad de Córdoba, Hospital Universitario Reina Sofía, 14004 Córdoba, Spain; 3 Instituto de Investigación Biomédica de Málaga-IBIMA, UGC Endocrinología y Nutrición, Hospital Regional Universitario de Málaga, 29009 Málaga, Spain

**Keywords:** cannabinoid, streptozotocin, phytocannabinoid, type 1 diabetes, endocannabinoid system, chronic kidney disease

## Abstract

Anti-inflammatory and antidiabetogenic properties have been ascribed to cannabidiol (CBD). CBD-based medicinal drugs have been approved for over a lustrum, and a boom in the commercialization of CBD products started in parallel. Herein, we explored the efficacy of CBD in streptozotocin (STZ)-induced diabetic mice to prevent diabetic nephropathy at onset. Eight-to-ten-week-old C57BL6J male mice were treated daily intraperitoneally with 10 mg/kg of CBD or vehicle for 14 days. After 8 days of treatment, mice were challenged with STZ or vehicle (healthy-control). At the end of the study, non-fasting blood glucose (FBG) level was 276 ± 42 mg/dL in vehicle-STZ-treated compared to 147 ± 9 mg/dL (*p* ≤ 0.01) in healthy-control mice. FBG was 114 ± 8 mg/dL in vehicle-STZ-treated compared to 89 ± 4 mg/dL in healthy-control mice (*p* ≤ 0.05). CBD treatment did not prevent STZ-induced hyperglycemia, and non-FBG and FBG levels were 341 ± 40 and 133 ± 26 mg/dL, respectively. Additionally, treatment with CBD did not avert STZ-induced glucose intolerance or pancreatic beta cell mass loss compared to vehicle-STZ-treated mice. Anatomopathological examination showed that kidneys from vehicle-STZ-treated mice had a 35% increase of glomerular size compared to healthy-control mice (*p* ≤ 0.001) and presented lesions with a 43% increase in fibrosis and T cell infiltration (*p* ≤ 0.001). Although treatment with CBD prevented glomerular hypertrophy and reduced T cell infiltration, it significantly worsened overall renal damage (*p* ≤ 0.05 compared to vehicle-STZ mice), leading to a more severe renal dysfunction than STZ alone. In conclusion, we showed that CBD could be detrimental for patients with type 1 diabetes, particularly those undergoing complications such as diabetic nephropathy.

## 1. Introduction

*Cannabis sativa* is one of the most cultivated plants over the years due to its high economical and medical value [1]. Numerous pharmacological studies have reported that *C. sativa* has a variety of properties, including analgesic [2], antibacterial [3], and anti-inflammatory [4] effects among others. The most abundant non-psychotropic cannabinoid from *Cannabis sativa* is cannabidiol (CBD), which has increased interest for medicinal applications because of its broad biological activity spectrum. The first study about the effectiveness of CBD as an anticonvulsant was carried out by Consroe and Wolking in 1977 [5], a starting point for further research that demonstrated preclinical-evidences of CDB such as anticonvulsant, antinausea, and analgesic effects [6]. Currently, CBD, alone (Epidiolex) or in combination with THC (Sativex), is approved in some countries for the treatment of refractory epilepsy in children and spasticity in multiple sclerosis, respectively [7]. Although the legal status is not clarified, CBD is also available as a dietary supplement.

CBD signals through several receptors, triggering the serotonin, opioid, and endocannabinoid systems (ECS) [8]. CBD is also a potent antioxidant and a negative allosteric modulator (NAM) of the cannabinoid type 1 receptor (CB1R) [9]. The ECS is an endogenous signal network with multiple functions under physiological and pathological conditions. The ECS consists of cannabinoid receptors, endogenous fatty acid ligands, and their biosynthetic and degradative enzymes. The main receptors of the ECS, the CB1R and the cannabinoid type 2 receptor (CB2R) are seven-transmembrane G protein-coupled receptors (GPCRs) widely distributed in different tissues [10]. CB1R is found in the CNS, in metabolically active tissues including the liver, endocrine pancreas, kidney, and immune cells, while CB2Rs are mainly distributed in the peripheral regions of the spleen and the tonsils and immune cells [11]. Both cannabinoid receptors are involved in the development of diabetic nephropathy, and while renal CB1R is overexpressed in diabetic nephropathy, CB2R is downregulated [12,13,14].

The pharmacological effect of CBD has been previously investigated for the treatment of a wide range of diseases, metabolic and autoimmune disorders, such as type 1 diabetes (T1D). CBD ameliorates the manifestation and delays the onset of T1D in non-obese diabetic mice (NOD) [15,16]. T1D is an autoimmune disease with no cure characterized by a progressive immune cell infiltration in and around the islets, which leads to the gradual loss of insulin-producing beta cells, hyperglycemia, and eventually, an absolute insulin deficiency [17]. High blood glucose due to T1D increases the risk of macro and microvascular complications such as nephropathy [18]. In rodents, blockade of CB1R protects insulin-producing pancreatic beta cells and prevents islet inflammation in obesity [14,19]. It also alleviates diabetic nephropathy in type 2 diabetic rats [14]. Additionally, we previously showed that a (+)-enantiomer of CBD ameliorated diabetic nephropathy at onset in mice [20]. However, despite the beneficial effects of CBD on T1D NOD mice described 15 years ago, its potential effect on diabetic nephropathy has not been investigated.

Herein we document the outcome of CBD treatment at the onset of diabetic nephropathy in a T1D mouse model and show that, overall, CBD has a detrimental impact on diabetic nephropathy.

## 2. Results

### 2.1. Cannabidiol Does Not Avert Streptozotocin-Induced Hyperglycemia and Glucose Intolerance

**Figure 1:**
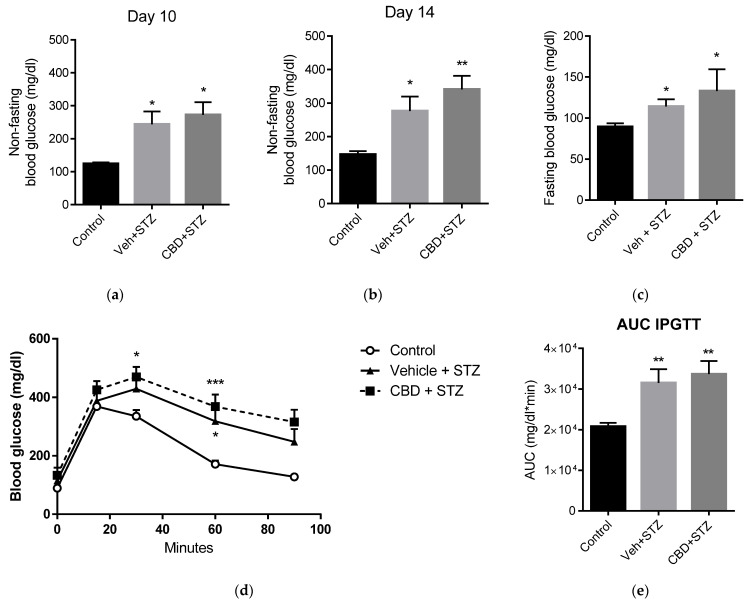
Cannabidiol (CBD) does not prevent streptozotocin (STZ)-induced diabetes. (**a**) Mid-study (3 days of STZ treatment) and (**b**) end-of-study (7 days of STZ treatment) non-fasting blood glucose levels in healthy control (control), vehicle-STZ- (Veh-STZ), and CBD-STZ-treated mice. (**c**) Fasting blood glucose at the end of the study. (**d**) Intraperitoneal glucose tolerance test (IPGTT) in vehicle-STZ and CBD-STZ-treated mice compared to control at the end of the study. (**e**) The area under the curve of the IPGTT was calculated using the trapezoidal rule. Values are expressed as mean ± SEM (*n* = 6–7 animals per group). * *p* < 0.05, ** *p* < 0.01, *** *p* < 0.001 vs. vehicle-citrate buffer-treated mice (control).

Eight-to-ten-week-old mice were treated intraperitoneally with CBD (10 mg/kg; the same dose used in our concurrent study with (+)-enantiomers of CBD [20]) or vehicle (DMSO:Tween-80:PBS) for 14 days. On day 7, mice were challenged with streptozotocin (STZ) or vehicle (Saline; control group) was initiated and blood glucose was monitored. Non-fasting blood glucose was elevated after 3 days of STZ treatment in both vehicle-STZ and CBD-STZ-treated mice compared to healthy controls (Figure 1a). On day 14, non-fasting blood glucose was 1.8-fold and 2.3-fold higher in vehicle-STZ and CBD-STZ-treated mice, respectively, compared to control (Figure 1b), and fasting blood glucose was 114 ± 8 mg/dL and 133 ± 26 mg/dL compared to 89 ± 4 mg/dL in healthy control mice (Figure 1c). On day 14 both, vehicle-STZ and CBD-STZ-treated mice were glucose intolerant as shown by significantly higher blood glucose than control mice upon intraperitoneal glucose tolerance test (Figure 1d,e). In sum, treatment with CBD did not prevent STZ-induced hyperglycemia and glucose intolerance.

### 2.2. Cannabidiol Does Not Protect Beta Cell Mass from STZ

**Figure 2:**
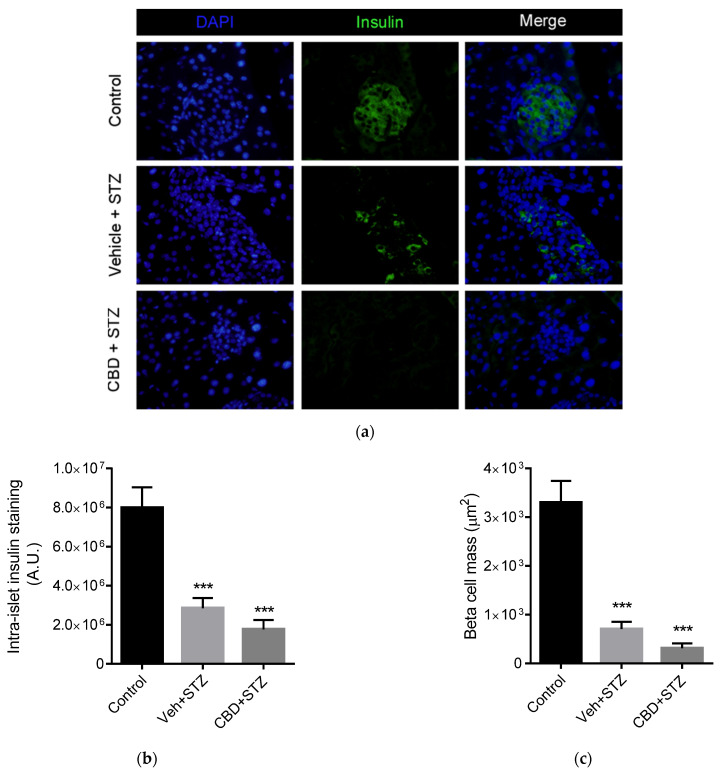
CBD does not prevent STZ-induced beta cell loss. (**a**) Representative images of immunofluorescent staining of insulin (green) and nuclei (DAPI, blue) of the pancreas from control, vehicle-STZ and CBD-STZ-treated mice (original magnification ×25, scale bar 50 μm). Quantification of (**b**) intra-islet staining and (**c**) beta cell mass (calculated as total beta cell area) per islet. *N* = 100 islets/group. Values are expressed as mean ± SEM (*n* = 6–7 animals per group). *** *p* < 0.001 vs. vehicle-citrate buffer-treated mice (control).

CBD has been shown to reduce insulitis in NOD/ShiLtJ mice, thus preserving beta cell mass [15]. We then investigated if CBD was able to protect beta cells from treatment with STZ. On day 15 of treatment, mice were sacrificed and the pancreas dissected and processed for immunohistochemistry. Insulin staining revealed that STZ induced a 2.8-fold reduction of insulin content, while treatment with CBD induced a 4.6-fold reduction of insulin content, compared to control (Figure 2a,b). Analysis of beta cell mass area showed that STZ alone induced a significant 4.8-fold reduction of beta cell mass, while treatment with CBD induced a 10-fold reduction of beta cell mass compared to control (Figure 2a,c). Overall, together with the data shown above, treatment with CBD did not avert STZ-induced diabetes in mice.

### 2.3. Treatment with CBD Worsens Renal Lesions in STZ-Treated Mice

**Figure 3:**
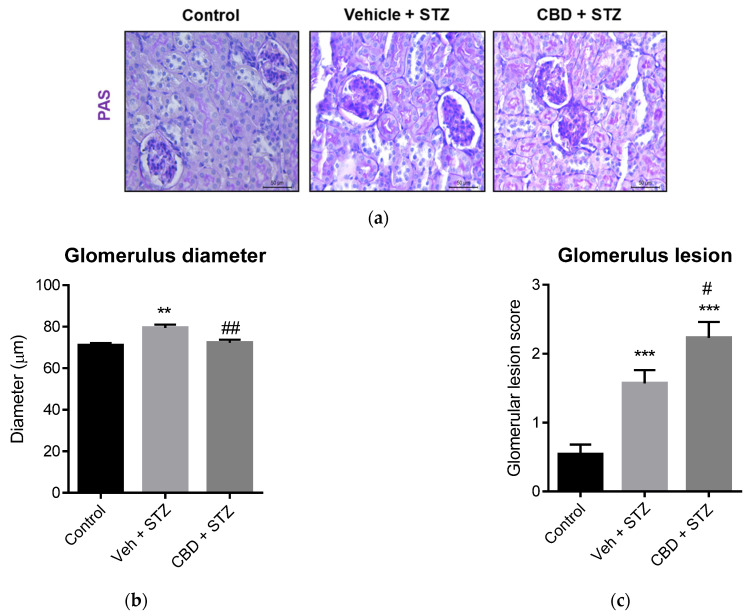
CBD worsens STZ-induced renal lesions in glomerulus. (**a**) Representative images of kidney stained with PAS from control, vehicle-STZ and CBD-STZ-treated mice (original magnification ×10, scale bar 50 μm). Quantification of (**b**) glomerulus diameter and (**c**) glomerular lesion score. Values are expressed as mean ± SEM (*n* = 6–7 animals per group). ** *p* < 0.01, *** *p* < 0.001 vs. vehicle-citrate buffer-treated mice (control); ^#^ *p* < 0.05, ^##^ *p* < 0.01 vs. vehicle-STZ.

**Figure 4:**
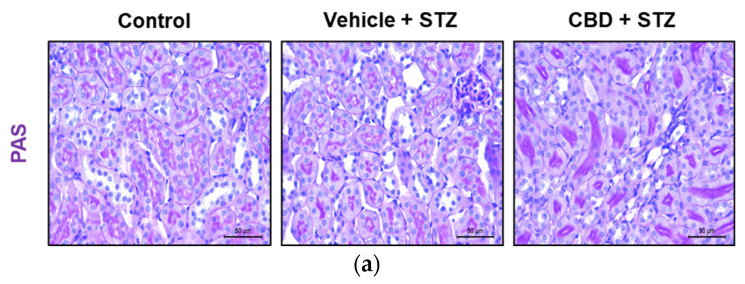

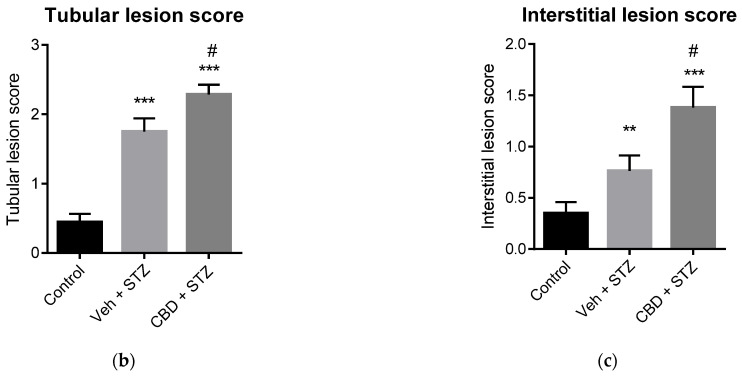
CBD worsens STZ-induced renal lesions in tubules and interstice. (**a**) Representative images of kidney stained with PAS from control, vehicle-STZ, and CBD-STZ-treated mice (original magnification ×10, scale bar 50 μm). Quantification of (**b**) tubular and (**c**) interstitial lesion score. Values are expressed as mean ± SEM (*n* = 6–7 animals per group). ** *p* < 0.01, *** *p* < 0.001 vs. vehicle-citrate buffer-treated mice (control); ^#^ *p* < 0.05 vs. vehicle-STZ.

Diabetic nephropathy is a severe complication of T1D, and previous studies showed that targeting the ECS ameliorates diabetic nephropathy in rodents [21]. Thus, we analyzed whether CBD was able to prevent STZ-induced renal lesions. Histochemical analysis of the kidney showed that STZ induced glomerular hypertrophy, as shown by a significant increase in glomerular size (Figure 3a,b), and glomerular lesions compared to control mice (Figure 3a,c). Although treatment with CBD did not increase the glomerular size (Figure 3a,b), it significantly increased glomerular lesions compared to control (Figure 3a,c). Moreover, treatment with CBD significantly worsened glomerular lesions compared to STZ alone (Figure 3a,c). Treatment with STZ also induced tubular (Figure 4a,b) and interstitial (Figure 4a,c) lesions, which included immune cell infiltration, compared to control. Compared to STZ, treatment with CBD significantly worsened tubular and interstitial lesions, with a patent infiltration of immune cells (Figure 4). Hence, anatomopathological analysis of the kidney revealed that CBD worsens the structural damages generated by STZ.

### 2.4. Treatment with CBD Partially Prevents STZ-Induced CD3^+^ Cells Infiltration in the Kidney

**Figure 5:**
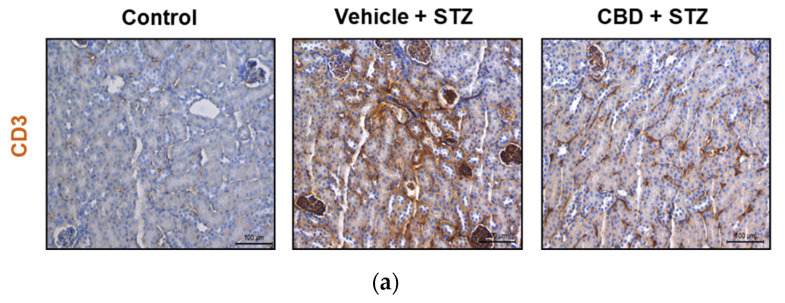

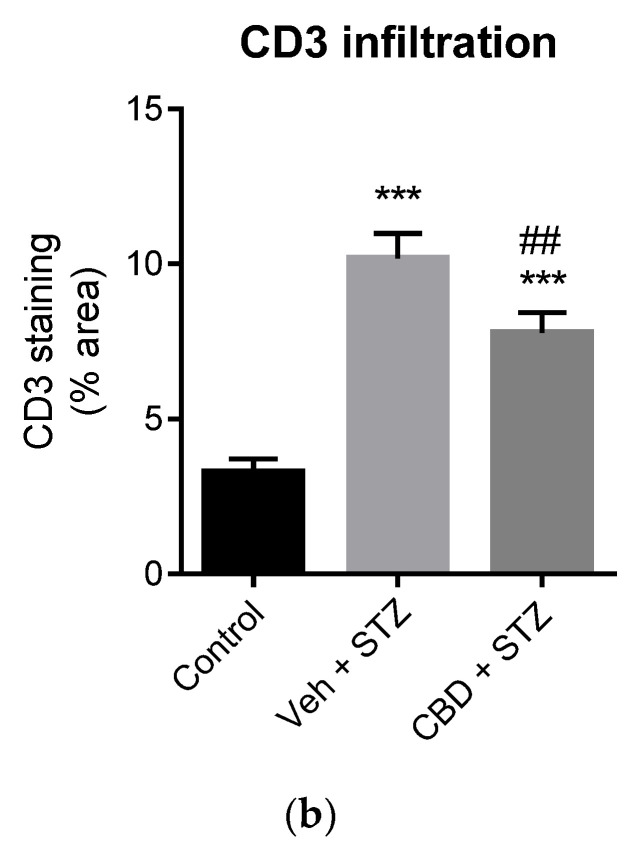
CBD significantly reduces but does not prevent STZ-induced immune cell infiltration in the kidney. (**a**) Representative images of immunostaining for CD3 cells in the kidney from control, vehicle-STZ, and CBD-STZ-treated mice (original magnification ×20, scale bar 100 μm). (**b**) Quantification of CD3 positive area (percentage of total kidney area). Values are expressed as mean ± SEM (*n* = 6–7 animals per group). *** *p* < 0.001 vs. vehicle-citrate buffer-treated mice (control); ^##^ *p* < 0.01 vs. vehicle-STZ.

Since we found an increase in renal lesions in both STZ and CBD-STZ-treated mice, we analyzed infiltration of immune T cells by immunohistochemistry of the kidneys using CD3 T cell marker. A significant increase was found in CD3^+^ T cells infiltration into the kidney of STZ-treated mice compared to control (Figure 5a,b). Treatment with CBD did not avert CD3^+^ T cell infiltration, although it was significantly lower by 1.3 folds compared to STZ-treated mice (Figure 5a,b). Thus, treatment with CBD had an anti-inflammatory effect despite the renal damages observed.

### 2.5. Treatment with CBD Does Not Avert STZ-Induced Renal Fibrosis and Worsens Renal Failure

**Figure 6:**
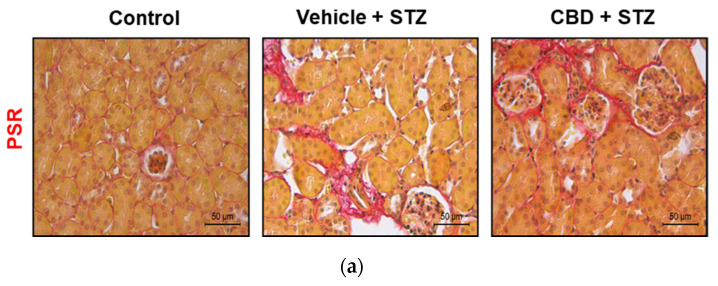

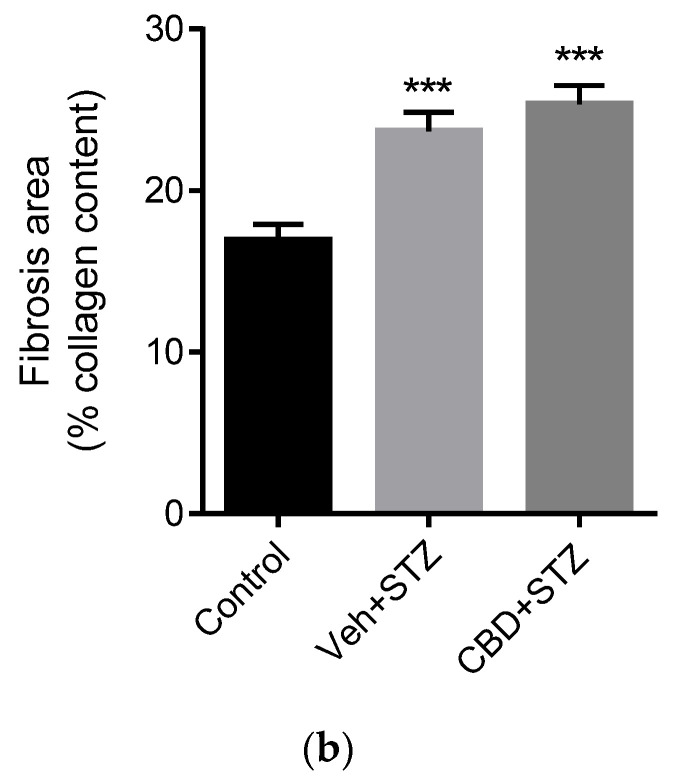
CBD does not avoid STZ-induced renal fibrosis. (**a**) Representative images of collagen staining of kidney from control, vehicle-STZ, and CBD-STZ-treated mice by picrosirius red dye (original magnification ×10, scale bar 50 μm). (**b**) Quantification of collagen positive area (expressed as a percentage of total kidney area). Values are expressed as mean ± SEM (*n* = 6–7 animals per group). *** *p* < 0.001 vs. vehicle-citrate buffer-treated mice (control).

**Figure 7:**
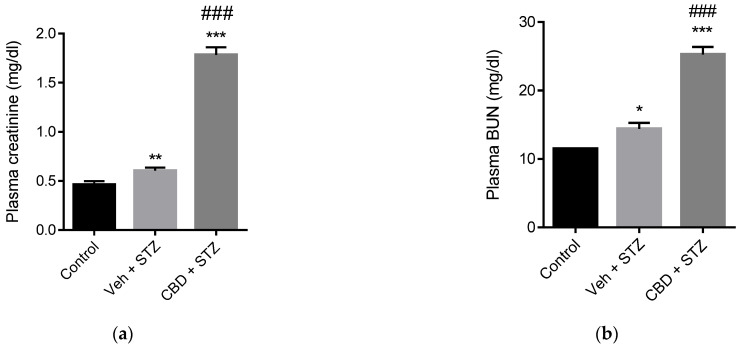
CBD worsens STZ-induced renal dysfunction. Quantification of (**a**) plasma creatinine and (**b**) blood urea nitrogen (BUN) in control, vehicle-STZ, and CBD-STZ-treated mice. Values are expressed as mean ± SEM (*n* = 6–7 animals per group). * *p* < 0.05, ** *p* < 0.01 and *** *p* < 0.001 vs. vehicle-citrate buffer-treated mice (control); ^###^ *p* < 0.001 vs. vehicle-STZ.

Glomerulosclerosis in advanced diabetic nephropathy is associated with end-stage kidney disease. We analyzed fibrosis in kidney samples from our mouse model. STZ significantly increased glomerular fibrosis compared to control mice of 1.4 folds (Figure 6a,b). Treatment with CBD did not prevent STZ-induced fibrosis, showing a 1.5-fold increase of the fibrotic area compared to control (Figure 6a,b). To assess renal function, we analyzed the levels of creatinine and blood urea nitrogen (BUN). Mice challenged with STZ exhibited a significant increase in creatinine and BUN levels compared to healthy control mice (Figure 7a,b). Treatment with CBD further increased creatinine (2.9-fold higher) and BUN levels (1.5-fold higher) compared to STZ alone (Figure 7a,b). Altogether, treatment with CBD had a synergistic effect with STZ, leading to a more severe renal dysfunction.

## 3. Discussion

CBD is a known anti-inflammatory and antidiabetogenic phytocannabinoid that has been approved for over a lustrum for use in specific medical conditions. Despite the commercial boom associated with its legalization, its effects on various pathologies and specific targets remain unexplored. Herein we investigated its potential effects in a mouse model at the onset of diabetic nephropathy. We found that CBD induced stronger changes than STZ alone, and worsened renal damage, showing that its use by patients with T1D could be potentially detrimental and further deteriorate renal function in those with diabetic nephropathy.

Some cannabinoid derivatives have been shown to increase resistance to hepatic steatosis and reversal of hepatic steatosis in Non-alcoholic Fatty liver disease (NAFLD) [22]. Studies from our group revealed that the treatment with the derivate Δ9-tetrahydrocannabinolic acid reduced body weight and adiposity, improved glucose tolerance, and attenuated liver fibrosis and immune cell infiltration in NAFLD rodent models [23]. Similarly, the synthetic cannabinoid Abn-CBD exerts beneficial immunomodulatory actions in the liver of obese prediabetic mice with NAFLD [24]. In the case of CBD, it has been reported that it attenuates alcohol-induced liver steatosis [25], although it could be due to its reported effect on reducing alcohol intake in mice [26]. Differences between CBD’s effect in the resolution of liver fibrosis in contrast to detrimental effects in renal function could also be due to tissues specific mechanisms.

CBD delays the occurrence of hyperglycemia in the autoimmune T1D NOD mice [15,16] but it did not prevent STZ-induced hyperglycemia nor protected beta cells from damage by STZ. Although CBD ameliorates T1D in NOD mice, Dr. Weiss and colleagues did not study its effect on the kidney [15,16]. In sham animals, CBD has shown to have no detrimental effects in the kidney compared to the vehicle-treated group [27] but in the case of acute kidney injury, CBD has been shown to protect from ischemia/reperfusion renal injury [27,28]. Few studies have shown cases of acute kidney injury in synthetic cannabinoids consumers [29,30]. However, the consumption of cannabis increases the risk of mild renal function decline [31]. Further epidemiological studies are required to clarify these discrepancies and unravel whether they are due to CBD alone or its combination with THC or other phytocannabinoids, or its combination with other drugs of abuse.

**Figure 8:**
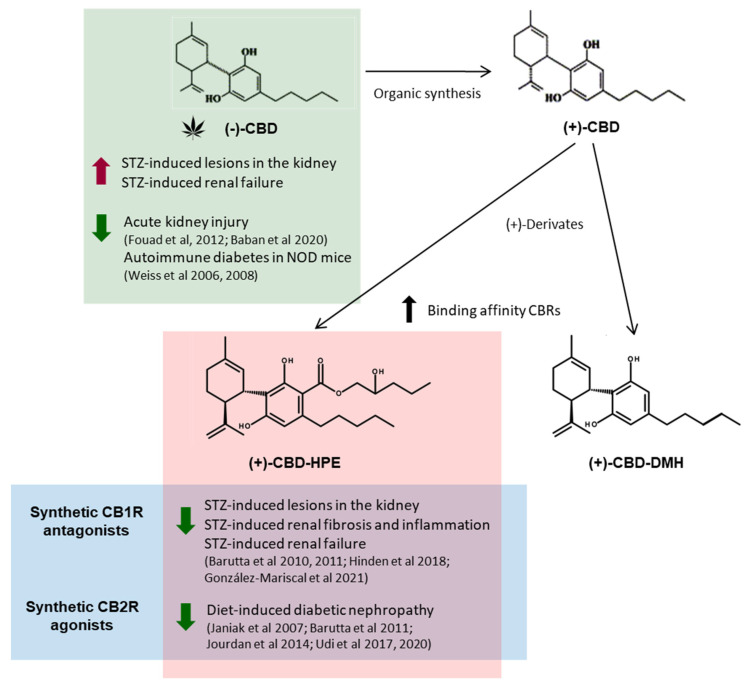
Summary of the action of the natural (phyto-) and synthetic cannabidiol (+)-derivatives on different models of diabetic nephropathy.

Overall, and despite the short-time treatment, CBD induced stronger changes in the kidney than STZ alone. Natural occurring CBD activates a plethora of systems including the ECS. Renal CB1R becomes overactivated in diabetic nephropathy, while, in opposition, renal CB2R is downregulated [12,13,14]. All of the naturally occurring CBD-type cannabinoids have a (−)-trans absolute configuration, corresponding to negative optical rotation [32], while (+)-CBD can only be obtained by organic synthesis. From (+)-trans-CBD, novel derivatives have been developed with enhancing binding affinity to CB1R and CB2R. Among them, (+)-Cannabidiol-dimethyl heptyl has shown analgesic activity [33,34]. Inverse agonists of CB1R have been shown to prevent diabetic nephropathy in rodent models [14]. We have recently described chemical modifications of (−)-CBD that increases the binding of CB1Rs and CB2Rs [20]. In the same way (−)-CBD-2-hydroxy pentyl ((−)-CBD-HPE) had a moderate binding to CB1R but strong for CB2R. In functional assays (−)-CBD-HPE behaved as an agonist for CB2R and antagonist for CB1R [35]. We synthesized the (+)-enantiomer of CBD and its derivative (+)-CBD hydroxy pentyl ester ((+)-CBD-HPE) and showed that (+)-CBD-HPE exhibited an enhanced CB1R and CB2R binding and CB1R antagonist/CB2R agonist functions compared to it respective (−) enantiomer [20]. Concurrently to CBD and at the same dose, (+)-CBD-HPE prevented STZ-induced lesions in the kidney and avoided renal fibrosis and CD3^+^T cell infiltration [20]. The beneficial effects obtained with (+)-CBD-HPE are likely due to its enhanced affinity and activity over the CB1R and CB2R compared to CBD. Interestingly, activation of CB2R protects against diet-induced diabetic nephropathy [36]. CB1R becomes overactivated in the kidney upon treatment with STZ [12] and extensive work by various groups have described that CB1R in the kidney plays a key role in the development of diabetic nephropathy, and its blockade is a promising therapy [12,14,21,37,38,39]. Specifically, in the STZ-induced diabetic model, AM251 (CB1R antagonist with IC50 = 8 nM and Ki = 7.49 nM) prevents STZ-induced loss of nephrin, podocin, and ZO-1, ameliorating albuminuria [12]. Thus, it is likely that CBD does not function as NAM for CB1R in the STZ model (Figure 8). Usage of opioids has been linked to the development of chronic kidney disease (CKD) [40], and CBD activates the opioid system, which could be partially responsible for CBD’s detrimental effect on the kidney.

One of the known properties of CBD besides analgesia is its anti-inflammatory capacity. Despite the increase in STZ-induced renal damage upon CBD treatment, we observed a slight reduction in CD3^+^ immune T cell infiltration, most likely due to its anti-inflammatory properties, in agreement with the previous findings in NOD mice [15,17]. However, it was not sufficient to prevent infiltration and eventual damage of the tissue. Nevertheless, its anti-inflammatory properties could be of benefit when diabetic nephropathy is already established and not at onset, as it has been previously suggested for other cannabinoids. In an animal model of already established diabetic nephropathy, long-term treatment with AM6545 and AM1241 had an anti-inflammatory role that reverted renal abnormalities at the structural and functional level [41]. Thus, its usage in patients at risk of developing CKD should be unadvisable in spite of its other potential benefits for CKD symptoms such as nausea, chronic pain, anorexia, and insomnia. Further studies using CBD in models with already established diabetic nephropathy to unveil its significance in this severe complication are warranted.

## 4. Materials and Methods

### 4.1. Animals

All experiments were performed in strict accordance with European Union (EU) and governmental regulations. Handling of animals was performed in compliance with the guidelines of the European Union Directive 2010/63/EU for the use and care of experimental animals; the Ethics Committee on Animal Experimentation of the University of Málaga (UMA, Málaga, Spain) and the Regional Government of Andalucía approved all the procedures described in this study (Project number 28/06/2018/107). Male C57BL/6 mice (Charles River France) were used in all experiments. Animals were housed in groups of 10 under controlled conditions of 12 h light/dark cycles at 20 °C (±2 °C) and 40–50% relative humidity, with free access to water and standard food. Eight- to 10-week-old C57BL6J male mice of 24.4 ± 0.2 g of body weight were randomized to 3 groups: healthy control (vehicle and citrate buffer), vehicle-streptozotocin (STZ), and CBD-STZ. Mice were injected daily intraperitoneally (i.p.) with vehicle (saline:DMSO:Tween-80, 95:4:1) or 10 mg/kg of CBD for 7 days. The dose of CBD was selected based on previous literature to have no effect on sham animals but to have a positive effect on other models of kidney injury [27], and it was equivalent to a concurrent study using 10 mg/kg of (+)-CBD-HPE that showed a positive outcome compared to STZ-Vehicle (protected against STZ-induced damage, fibrosis and inflammation in the kidney) [20]. Mice were fasted for 4 h prior i.p. injections with STZ or citrate buffer (healthy controls) as described previously [42]. Mice were given 10% sucrose water for 48 h upon STZ treatment to prevent ongoing toxicity and to avoid hypoglycemia. Blood glucose was monitored daily using the OneTouch Ultra blood glucose meter (LifeScan IP Holdings, LLC, Malvern, PA, USA). After 7 days mice were euthanized by cervical dislocation, and tissues and blood were collected and processed immediately for histological and biochemical analysis. *n* = 6–7 animals/group.

### 4.2. Intraperitoneal Glucose Tolerance Test

Mice were fasted overnight and given free access to water. Mice were given i.p. a bolus of 2 g/kg glucose and blood glucose was determined at 0, 15, 30, 60, and 90 min.

### 4.3. Histochemical and Immunohistochemistry Analysis

The pancreas and kidney were dissected and fixed in methanol-free 4% paraformaldehyde (Pierce) for 6 h at room temperature or 24 h at 4 °C, respectively, before paraffin embedding. Kidney sections (5 μm) were deparaffinized and dehydrated in a graded series (100–70%) of ethanol washes and stained with Periodic Acid Schiff (PAS) (Sigma-Aldrich, St. Louis, MO, USA) to evaluate renal pathology. Two independent assessors reviewed histological sections in a blinded manner and graded (0–3 scale) glomerular changes (hypercellularity, mesangial expansion, and capillary dilation, 40 glomeruli), tubular lesions (atrophy and degeneration, 20 fields at 40× magnification), and interstitial damage (fibrosis and inflammation, 20 fields at 40× magnification) [43]. Imaging was performed using a light microscope Leica DM2000 microscope. Glomerular area and diameter were marked manually and calculated automatically using Image J software (http://rsb.info.nih.gov/.ij; 1.52p, accessed on 22 June 2019). Kidney collagen was detected by Picrosirius Red staining (PSR) following the manufacturer’s instructions (Sigma-Aldrich). Quantitative evaluation of PSR staining was estimated as the staining under a grid intersection/total number of intersections multiplied by 100 (% of the fraction area), as described previously [44]. The data were represented by the area percentage of each slide positive (20 fields at 40× magnification) for red stain which was calculated using Image J software. The mean scores were calculated by mouse and by group. For immunohistochemistry, slides were deparaffinized and rehydrated, and antigen retrieval was performed in 10 mM sodium citrate buffer (pH 6) at 95 °C for 10 min. To block endogenous peroxidase activity, sections were immersed in 3.3% hydrogen peroxide in methanol for 30 min and blocked for 1 h with blocking solution (Merck-Millipore, Burlington, MS, USA) at room temperature. T lymphocytes were detected with anti-CD3 primary antibody (SC-20047, Santa Cruz Biotechnology, Quimigen, Madrid, Spain; 1:50) overnight at 4 °C. Then, the slides were incubated for 1 h at room temperature with the biotin-conjugated secondary antibody (goat anti-mouse, 21,538, Merck-Millipore). The reaction product was detected by avidin-biotin-peroxidase (Vector Laboratories, Palex Medical, Barcelona, Spain), the color reaction was developed with DAB (3,3′Diaminobenzidine) chromogen (Dako, Santa Clara, CA, USA) and subsequent counter-stained with hematoxylin. DAB staining was quantified using Image J Fiji after color deconvolution and further processed by Image J software to quantify signal intensity. The data were represented by the area percentage of each slide positive for red or blue stain. Pancreas sections were deparaffinized and boiled for 10 min in sodium citrate buffer 10 mM. Sections were blocked in 5% goat serum 0.3% Triton-X-100 in PBS for 30 min at 37 °C before incubation with the primary antibody in 1% serum, 0.3% Triton-X-100 in PBS overnight at 4 °C. Primary antibody used was mouse anti-insulin (Sigma I-2018; 1:500). Sections were washed 3 times for 5 min in 0.3% Triton-X-100 PBS and incubated with a secondary antibody in 1% serum, 0.3% Triton-X-100 in PBS for 30 min at 37 °C and then washed 3 times for 5 min in 0.3% Triton-X-100 PBS. Alexa Fluor antibodies were incubated for 45 min at 37 °C, and nuclei stained using DAPI (Vector Laboratories) for immunofluorescence. All images were acquired at 20× using an Olympus BX41 and analyzed using Image J software 1.52p.

### 4.4. Blood Urea Nitrogen Analysis

Levels of blood urea nitrogen (BUN) were determined in plasma using the DetectX^®^ Urea Nitrogen (BUN) Colorimetric Detection Kit from Arbor Assays (Quimigen, Madrid, Spain) following manufacturer instructions. Samples were diluted 1:10–1:20 and run in duplicates.

### 4.5. Plasma Creatinine Analysis

Creatinine was quantified in plasma using the DetectX^®^ Serum Creatinine Detection Kit from Arbor Assays (Quimigen, Madrid, Spain) following manufacturer instructions. Samples were diluted 1:2 and run in duplicates.

### 4.6. Data and Statistical Analysis

In vivo data are mean ± SEM. One-way analysis of variance (ANOVA) followed by Tukey’s post-hoc test for parametric analysis or Kruskal-Wallis post-hoc test for non-parametric analysis were used to determine the statistical significance. The level of significance was set at *p* < 0.05. Statistical analyses were performed using GraphPad Prism version 8.00 (GraphPad, San Diego, CA, USA).

## 5. Conclusions

Despite its anti-inflammatory properties, we have found that CBD worsens diabetic nephropathy at onset and leads to earlier end-stage kidney disease in our mouse model. CBD-based medicinal drugs have been approved for over a lustrum, and a boom in the commercialization of CBD products started in parallel. Thus, caution should be taken by patients with T1D and those undergoing complications such as diabetic nephropathy.

## Data Availability

Data is contained within the article.

## References

[1] Li H., Liu Y., Tian D., Tian L., Ju X., Qi L., Wang Y., Liang C. (2020). Overview of cannabidiol (CBD) and its analogues: Structures, biological activities, and neuroprotective mechanisms in epilepsy and Alzheimer’s disease. Eur. J. Med. Chem..

[2] Pertwee R.G., Gibson T.M., A Stevenson L., A Ross R., Banner W.K., Saha B., Razdan R.K., Martin B.R. (2000). O-1057, a potent water-soluble cannabinoid receptor agonist with antinociceptive properties. Br. J. Pharmacol..

[3] Appendino G., Gibbons S., Giana A., Pagani A., Grassi G., Stavri M., Smith E., Rahman M. (2008). Antibacterial Cannabinoids from Cannabis sativa: A Structure−Activity Study. J. Nat. Prod..

[4] Liu W.M., Fowler D.W., Dalgleish A.G. (2010). Cannabis-derived substances in cancer therapy--an emerging anti-inflammatory role for the cannabinoids. Curr. Clin. Pharmacol..

[5] Consroe P., Wolkin A. (1977). Cannabidiol—Antiepileptic drug comparisons and interactions in experimentally induced seizures in rats. J. Pharmacol. Exp. Ther..

[6] Massi P., Solinas M., Cinquina V., Parolaro D. (2013). Cannabidiol as potential anticancer drug. Br. J. Clin. Pharmacol..

[7] Fiani B., Sarhadi K.J., Soula M., Zafar A., Quadri S.A. (2020). Current application of cannabidiol (CBD) in the management and treatment of neurological disorders. Neurol. Sci..

[8] Zou S., Kumar U. (2018). Cannabinoid Receptors and the Endocannabinoid System: Signaling and Function in the Central Nervous System. Int. J. Mol. Sci..

[9] Tham M., Yilmaz O., Alaverdashvili M., Kelly M.E.M., Denovan-Wright E.M., LaPrairie R.B. (2019). Allosteric and orthosteric pharmacology of cannabidiol and cannabidiol-dimethylheptyl at the type 1 and type 2 cannabinoid receptors. Br. J. Pharmacol..

[10] Sviženska I., Dubový P., Sulcova A. (2008). Cannabinoid receptors 1 and 2 (CB1 and CB2), their distribution, ligands and functional involvement in nervous system structures—A short review. Pharmacol. Biochem. Behav..

[11] Izzo A.A., Sharkey K. (2010). Cannabinoids and the gut: New developments and emerging concepts. Pharmacol. Ther..

[12] Barutta F., Corbelli A., Mastrocola R., Gambino R., Di Marzo V., Pinach S., Rastaldi M.P., Perin P.C., Gruden G. (2010). Cannabinoid Receptor 1 Blockade Ameliorates Albuminuria in Experimental Diabetic Nephropathy. Diabetes.

[13] Barutta F., Piscitelli F., Pinach S., Bruno G., Gambino R., Rastaldi M.P., Salvidio G., Di Marzo V., Perin P.C., Gruden G. (2011). Protective Role of Cannabinoid Receptor Type 2 in a Mouse Model of Diabetic Nephropathy. Diabetes.

[14] Jourdan T., Szanda G., Rosenberg A.Z., Tam J., Earley B.J., Godlewski G., Cinar R., Liu Z., Liu J., Ju C. (2014). Overactive cannabinoid 1 receptor in podocytes drives type 2 diabetic nephropathy. Proc. Natl. Acad. Sci. USA.

[15] Weiss L., Zeira M., Reich S., Har-Noy M., Mechoulam R., Slavin S., Gallily R. (2006). Cannabidiol lowers incidence of diabetes in non-obese diabetic mice. Autoimmunity.

[16] Weiss L., Zeira M., Reich S., Slavin S., Raz I., Mechoulam R., Gallily R. (2008). Cannabidiol arrests onset of autoimmune diabetes in NOD mice. Neuropharmacology.

[17] Jacobsen L., Schatz D. (2016). Current and future efforts toward the prevention of type 1 diabetes. Pediatr. Diabetes.

[18] Forouhi N., Wareham N.J. (2014). Epidemiology of diabetes. Medicine.

[19] González-Mariscal I., Montoro R., Doyle M.E., Liu Q.-R., Rouse M., O’Connell J.F., Calvo S.S.-C., Krzysik-Walker S.M., Ghosh S., Carlson O.D. (2018). Absence of cannabinoid 1 receptor in beta cells protects against high-fat/high-sugar diet-induced beta cell dysfunction and inflammation in murine islets. Diabetology.

[20] González-Mariscal I., Carmona-Hidalgo B., Winkler M., Unciti-Broceta J.D., Escamilla A., Gómez-Cañas M., Fernández-Ruiz J., Fiebich B.L., Romero-Zerbo S.-Y., Bermúdez-Silva F.J. (2021). (+)-trans-Cannabidiol-2-hydroxy pentyl is a dual CB1R antagonist/CB2R agonist that prevents diabetic nephropathy in mice. Pharmacol. Res..

[21] Hinden L., Udi S., Drori A., Gammal A., Nemirovski A., Hadar R., Baraghithy S., Permyakova A., Geron M., Cohen M. (2017). Modulation of Renal GLUT2 by the Cannabinoid-1 Receptor: Implications for the Treatment of Diabetic Nephropathy. J. Am. Soc. Nephrol..

[22] Dibba P., Li A., Cholankeril G., Iqbal U., Gadiparthi C., Khan M.A., Kim D., Ahmed A. (2018). Mechanistic Potential and Therapeutic Implications of Cannabinoids in Nonalcoholic Fatty Liver Disease. Medicines.

[23] Carmona-Hidalgo B., González-Mariscal I., García-Martín A., Prados M.E., Ruiz-Pino F., Appendino G., Tena-Sempere M., Muñoz E. (2021). Δ9-Tetrahydrocannabinolic Acid markedly alleviates liver fibrosis and inflammation in mice. Phytomedicine.

[24] Romero-Zerbo S.Y., García-Fernández M., Espinosa-Jiménez V., Pozo-Morales M., Escamilla-Sánchez A., Sánchez-Salido L., Lara E., Cobo-Vuilleumier N., Rafacho A., Olveira G. (2020). The Atypical Cannabinoid Abn-CBD Reduces Inflammation and Protects Liver, Pancreas, and Adipose Tissue in a Mouse Model of Prediabetes and Non-alcoholic Fatty Liver Disease. Front. Endocrinol..

[25] Wang Y., Mukhopadhyay P., Cao Z., Wang H., Feng D., Haskó G., Mechoulam R., Gao B., Pacher P. (2017). Cannabidiol attenuates alcohol-induced liver steatosis, metabolic dysregulation, inflammation and neutrophil-mediated injury. Sci. Rep..

[26] Viudez-Martínez A., García-Gutiérrez M.S., Navarron C., Morales-Calero M.I., Navarrete F., Torres-Suárez A.I., Manzanares J. (2018). Cannabidiol reduces ethanol consumption, motivation and relapse in mice. Addict. Biol..

[27] Baban B., Khodadadi H., Vaibhav K., Marchetti C., Riccardi C., Mozaffari M.S. (2020). Regulation of Innate Lymphoid Cells in Acute Kidney Injury: Crosstalk between Cannabidiol and GILZ. J. Immunol. Res..

[28] Fouad A.A., Al-Mulhim A.S., Jresat I. (2012). Cannabidiol treatment ameliorates ischemia/reperfusion renal injury in rats. Life Sci..

[29] Bhanushali G.K., Jain G., Fatima H., Leisch L.J., Thornley-Brown D. (2012). AKI Associated with Synthetic Cannabinoids: A Case Series. Clin. J. Am. Soc. Nephrol..

[30] Kazory A., Aiyer R. (2013). Synthetic marijuana and acute kidney injury: An unforeseen association. Clin. Kidney J..

[31] Vupputuri S., Batuman V., Muntner P., Bazzano L.A., Lefante J.J., Whelton P.K., He J. (2004). The risk for mild kidney function decline associated with illicit drug use among hypertensive men. Am. J. Kidney Dis..

[32] Vollner L., Bieniek D., Korte F. (1969). Haschisch XX: Cannabidivarin, ein neuer Haschisch-Inhaltsstoff. Tetrahedron Lett..

[33] Fride E., Feigin C., Ponde D.E., Breuer A., Hanuš L., Arshavsky N., Mechoulam R. (2004). (+)-Cannabidiol analogues which bind cannabinoid receptors but exert peripheral activity only. Eur. J. Pharmacol..

[34] Tchilibon S., Ponde D.E., Breuer A., Fride E., Mechoulam R., Hanuš L.O. (2005). Enantiomeric cannabidiol derivatives: Synthesis and binding to cannabinoid receptors. Org. Biomol. Chem..

[35] Götz M.R., Collado J.A., Fernández-Ruiz J., Fiebich B.L., García-Toscano L., Gómez-Cañas M., Koch O., Leha A., Muñoz E., Navarrete C.M. (2019). Structure–Effect Relationships of Novel Semi-Synthetic Cannabinoid Derivatives. Front. Pharmacol..

[36] A Jenkin K., O’Keefe L., Simcocks A.C., Briffa J.F., Mathai M.L., McAinch A., Hryciw D. (2015). Renal effects of chronic pharmacological manipulation of CB 2 receptors in rats with diet-induced obesity. Br. J. Pharmacol..

[37] Janiak P., Poirier B., Bidouard J.-P., Cadrouvele C., Pierre F., Gouraud L., Barbosa I., Dedio J., Maffrand J.-P., Le Fur G. (2007). Blockade of cannabinoid CB1 receptors improves renal function, metabolic profile, and increased survival of obese Zucker rats. Kidney Int..

[38] Udi S., Hinden L., Earley B., Drori A., Reuveni N., Hadar R., Cinar R., Nemirovski A., Tam J. (2017). Proximal Tubular Cannabinoid-1 Receptor Regulates Obesity-Induced CKD. J. Am. Soc. Nephrol..

[39] Udi S., Hinden L., Ahmad M., Drori A., Iyer M.R., Cinar R., Herman-Edelstein M., Tam J. (2020). Dual inhibition of cannabinoid CB 1 receptor and inducible NOS attenuates obesity-induced chronic kidney disease. Br. J. Pharmacol..

[40] Mallappallil M., Sabu J., Friedman E.A., Salifu M. (2017). What Do We Know about Opioids and the Kidney?. Int. J. Mol. Sci..

[41] Barutta F., Grimaldi S., Gambino R., Vemuri K., Makriyannis A., Annaratone L., Di Marzo V., Bruno G., Gruden G. (2017). Dual therapy targeting the endocannabinoid system prevents experimental diabetic nephropathy. Nephrol. Dial. Transplant..

[42] Deeds M.C., Anderson J.M., Armstrong A.S., A Gastineau D., Hiddinga H.J., Jahangir A., Eberhardt N.L., Kudva Y.C. (2011). Single dose streptozotocin-induced diabetes: Considerations for study design in islet transplantation models. Lab. Anim..

[43] Recio C., Lazaro I., Oguiza A., Lopez-Sanz L., Bernal S., Blanco J., Egido J., Gomez-Guerrero C. (2017). Suppressor of Cytokine Signaling-1 Peptidomimetic Limits Progression of Diabetic Nephropathy. J. Am. Soc. Nephrol..

[44] Hewitson T.D., Smith E.R., Samuel C.S. (2014). Qualitative and quantitative analysis of fibrosis in the kidney. Nephrology.

